# The genome organization of *Neurospora crassa* at high resolution uncovers principles of fungal chromosome topology

**DOI:** 10.1093/g3journal/jkac053

**Published:** 2022-03-04

**Authors:** Sara Rodriguez, Ashley Ward, Andrew T Reckard, Yulia Shtanko, Clayton Hull-Crew, Andrew D Klocko

**Affiliations:** Department of Chemistry & Biochemistry, University of Colorado Colorado Springs, Colorado Springs, CO 80918, USA; Department of Chemistry & Biochemistry, University of Colorado Colorado Springs, Colorado Springs, CO 80918, USA; Department of Chemistry & Biochemistry, University of Colorado Colorado Springs, Colorado Springs, CO 80918, USA; Department of Chemistry & Biochemistry, University of Colorado Colorado Springs, Colorado Springs, CO 80918, USA; Department of Chemistry & Biochemistry, University of Colorado Colorado Springs, Colorado Springs, CO 80918, USA; Department of Chemistry & Biochemistry, University of Colorado Colorado Springs, Colorado Springs, CO 80918, USA

**Keywords:** in situ Hi-C, Neurospora, genome organization, genome topology, chromatin

## Abstract

The eukaryotic genome must be precisely organized for its proper function, as genome topology impacts transcriptional regulation, cell division, replication, and repair, among other essential processes. Disruptions to human genome topology can lead to diseases, including cancer. The advent of chromosome conformation capture with high-throughput sequencing (Hi-C) to assess genome organization has revolutionized the study of nuclear genome topology; Hi-C has elucidated numerous genomic structures, including chromosomal territories, active/silent chromatin compartments, Topologically Associated Domains, and chromatin loops. While low-resolution heatmaps can provide important insights into chromosomal level contacts, high-resolution Hi-C datasets are required to reveal folding principles of individual genes. Of particular interest are high-resolution chromosome conformation datasets of organisms modeling the human genome. Here, we report the genome topology of the fungal model organism *Neurospora crassa* at a high resolution. Our composite Hi-C dataset, which merges 2 independent datasets generated with restriction enzymes that monitor euchromatin (*Dpn*II) and heterochromatin (*Mse*I), along with our *Dpn*II/*Mse*I double digest dataset, provide exquisite detail for both the conformation of entire chromosomes and the folding of chromatin at the resolution of individual genes. Within constitutive heterochromatin, we observe strong yet stochastic internal contacts, while euchromatin enriched with either activating or repressive histone post-translational modifications associates with constitutive heterochromatic regions, suggesting intercompartment contacts form to regulate transcription. Consistent with this, a strain with compromised heterochromatin experiences numerous changes in gene expression. Our high-resolution Neurospora Hi-C datasets are outstanding resources to the fungal community and provide valuable insights into higher organism genome topology.

## Introduction 

Eukaryotic genome topology, or the spatial organization of chromosomal DNA within the nucleus, plays a critical role in genome function, as DNA folding has roles in gene expression, epigenetics, the cell cycle, DNA replication and repair, development, and countless other DNA-templated processes (Cremer and Cremer 2001; Misteli 2007; Dekker 2008; Ong and Corces 2011; Bonev and Cavalli 2016; Yu and Ren 2017; Furlong and Levine 2018; Falk *et al.* 2019). Of these, genome topology arguably impacts transcriptional regulation to the greatest extent, yet research is only beginning to illuminate how long-range contacts impact gene expression. For proper levels of transcription in higher eukaryotes, enhancer and/or silencer regulatory elements, which can be thousands of basepairs (bp) distant on the linear chromosome, associate in close proximity with their cognate promoter within the 3D spatial organization of the nucleus (Tolhuis *et al.* 2002; West and Fraser 2005; Dekker 2008; Schoenfelder *et al.* 2015). Further, genes in the same biological pathway can spatially colocalize in RNA Pol II transcriptional hubs for coordinated, temporal control of gene expression (Chambeyron and Bickmore 2004; Ragoczy *et al.* 2006; Schoenfelder *et al.* 2010, 2015; Vieux-Rochas *et al.* 2015). Aberrant DNA organization that disrupts normal genome function can lead to disease in humans. Both abnormal chromosomal numbers resulting from chromosomal segregation defects, such as the trisomy of chromosome 21 causing Down’s syndrome (Gardiner and Davisson 2000), as well as more-subtle disruptions to genome topology, can drastically impact normal genome function. For example, a single genome rearrangement that disrupts a topological boundary can improperly allow the promoter of an oncogene to spatially associate with an enhancer, thereby stimulating oncogenic growth (Flavahan *et al.* 2016; Yu and Ren 2017; Dixon *et al.* 2018; Ghavi-Helm *et al.* 2019; Greenwald *et al.* 2019). Given the critical link between genome topology and function, in-depth studies of genome organization in both humans and more simplistic model organisms are essential.

Historically, genome topology was examined microscopically using electron or fluorescence microscopy, the latter using Fluorescent In Situ Hybridization (FISH) to label individual DNA sequences or fluorescently tagged proteins binding to specific genomic features (e.g. the centromeres) (Cremer and Cremer 2001). These techniques contributed to numerous scientific advancements regarding the organization of chromatin—the complex of DNA and proteins critical for genome structure and function—including the condensation of mitotic chromosomes, the formation of individual chromosome territories, the association of compacted, silent heterochromatin with the nuclear periphery while the decompressed, transcriptionally active euchromatin typically localizes to the center of the nucleus, and the colocalization of certain chromosomal features in some organisms, such as telomeres, which independently cluster yet are segregated from centromere clusters (Franke 1974; Speicher and Carter 2005; Zuleger *et al.* 2011; Padeken and Heun 2014; Klocko *et al.* 2016; Falk *et al.* 2019; Hoencamp *et al.* 2021). The advent of chromosome conformation capture (3C) and its subsequent coupling to high-throughput sequencing (Hi-C) revolutionized the study of genome topology: these exquisite procedures capture interacting genomic loci as individual DNA ligation products. Early reports examined genome topology across cell stages, in single cells, for inactivated X-chromosomes, and even between individual genes (Dekker *et al.* 2002; Lieberman-Aiden *et al.* 2009; Nora *et al.* 2012; Jin *et al.* 2013; Naumova *et al.* 2013; Rao *et al.* 2014; Nagano *et al.* 2017). Many discoveries regarding eukaryotic genome organization have been reported, or confirmed, using Hi-C data. These include metazoan chromosomes occupying individual territories in the nucleus (Imakaev *et al.* 2012; Sexton *et al.* 2012; Hoencamp *et al.* 2021), active euchromatin and silent heterochromatin forming segregated compartments (Lieberman-Aiden *et al.* 2009; Rao *et al.* 2014), and Topologically Associated Domains (TADs)—in which internal chromatin is more apt to interact than DNA outside TAD boundaries—structurally organizing the genome over Megabases of DNA (Nora *et al.* 2012; Sexton *et al.* 2012; Szabo *et al.* 2020). While lower resolution Hi-C datasets monitor chromatin interactions on (sub-)chromosomal scales, only high-resolution datasets with deep sequencing coverage have enough detail to scrutinize individual gene folding.

Rao *et al.* reported the gold-standard high-resolution Hi-C dataset of human cells to capture and elucidate novel genomic structures (Rao *et al.* 2014). This study was the first to introduce in situ Hi-C ligation, in which Hi-C ligation products are formed in the nucleus, rather than by large-volume proximity ligation, to more accurately capture genomic contacts. Further, Rao *et al.* presented contact matrices at an astonishing 1 kilobase (kb) resolution: interactions could be observed across the human genome between any two 1,000 basepair segments (“bins”; in Hi-C datasets, chromosomal DNA is divided into nonoverlapping bins of a uniform size, and the array of bins in a contact matrix reports the interaction value between 2 bins for all bins across a genome). This work defined “resolution” as the smallest bin size at which 80% of all genomic loci (not all bins) have ≥1,000 contacts with any other locus, thereby providing outstanding structural detail of local contacts. Indeed, Rao *et al.* detailed 6 novel chromatin compartments (2 active “A” euchromatic and 4 silent “B” heterochromatic compartments) defined by specific epigenetic histone marks, nuclear lamina interactions, and cell cycle stages (Rao *et al.* 2014). Chromatin loops were also observed, where 2 distant loci, bound by a CTCF dimer, strongly interact to form a loop anchor (Rao *et al.* 2014). Later work highlighted roles of the protein complexes cohesin and condensin for the formation of loops and chromosomal territories; cohesin extrudes DNA to form loops (Rao *et al.* 2017; Yatskevich *et al.* 2019; Hoencamp *et al.* 2021). While these advances have been critical to discern human genome topology, the large size of the diploid human genome (∼6.6 × 10^9^ basepairs) and its complex underlying pathways make mechanistic studies of human genome organization difficult. Thus, further study of genome topology in simple model organisms can provide additional insight into eukaryotic chromosome organization; if Hi-C is to be used to dissect genome topology, high-resolution datasets are imperative.

The filamentous fungus *Neurospora crassa* is an outstanding model system for chromosome conformation studies (Galazka *et al.* 2016; Klocko *et al.* 2016; Courtney *et al.* 2020). Its relatively small, haploid genome (4.1 × 10^7^ basepairs in length, which is ∼167x smaller than the diploid human genome; note most fungi are predominantly haploid except for a short time during the sexual cycle when their nuclei are diploid, which differs from the predominantly diploid human cells) compacts similarly as DNA in higher organisms: the ratio of genome size to nuclear volume in fungi (∼9.8 × 10^6^ bp/μm^3^) mirrors that of some human cells (∼1.3 × 10^7^ bp/μm^3^), and TADs reportedly form across the Neurospora genome (Galazka *et al.* 2016). Further, the Neurospora genome is partitioned into euchromatin and heterochromatin (Galagan *et al.* 2003; Lewis *et al.* 2009; Galazka *et al.* 2016). Neurospora heterochromatin is homologous to that in humans: it is divided into constitutive and facultative subtypes. Permanently silent constitutive heterochromatin is found at gene-poor, repetitive, AT-rich DNA sequences and is post-translationally marked by methylation of cytosines in DNA and tri-methylation of lysine 9 on histone H3 (H3K9me3; catalyzed by the DIM-5/KMT-1 histone methyltransferase [HMTase] and bound by Heterochromatin Protein-1 [HP1]) (Tamaru *et al.* 2003; Freitag *et al.* 2004; Lewis *et al.* 2009, 2010; Klocko *et al.* 2015; Freitag 2017; Courtney *et al.* 2020). In contrast, temporarily silent facultative heterochromatin covering gene-rich regions is marked by post-translational di- or tri-methylation of lysine 27 on histone H3 (H3K27me2/3; catalyzed by the SET-7/KMT-6 HMTase) and/or di-methylation of lysine 36 on H3 (H3K36me2; catalyzed by the ASH1L HMTase) (Jamieson *et al.* 2013, 2016; Basenko *et al.* 2015; Freitag 2017; Bicocca *et al.* 2018; Courtney *et al.* 2020). Euchromatin in Neurospora is demarcated by post-translational di- or tri-methylation of lysine 4 on H3, tri-methylation of lysine 36 on H3 (catalyzed by the SET-2 HMTase), and acetylation of N-terminal tails on histone H3 and H4, among others (Adhvaryu *et al.* 2005; Lewis *et al.* 2009; Smith *et al.* 2010; Honda *et al.* 2012; Cemel *et al.* 2017; Bicocca *et al.* 2018; Zhu *et al.* 2019). All told, the genetically tractable *N.* *crassa* is an excellent and cost-efficient model of the human genome: high-resolution fungal in situ Hi-C datasets, using fewer Illumina sequencing reads for deep coverage of chromatin contacts across the genome, can elucidate fundamental principles of chromosome conformation inherent to eukaryotes.

Here, we present high-resolution in situ Hi-C datasets of the wild-type *N.* *crassa* genome that effectively monitor both euchromatin and heterochromatin, thereby illuminating chromosomal topology across the entire Neurospora genome. We independently generated datasets that monitor contacts in active and silent chromatin using common restriction enzymes with 4-base recognition sequences: *Dpn*II (^GATC) for euchromatin and *Mse*I (T^TAA) for heterochromatin; the latter facilitated the creation of a new Neurospora reference genome version, where we placed unassigned Supercontigs 8–20 in heterochromatic regions on chromosomes [Linkage Groups (LG)] I and V. By either merging the individual *Dpn*II and *Mse*I fastq files and building a single Hi-C contact matrix or generating a *Dpn*II/*Mse*I double digest in situ Hi-C library, we can assess the conformation of individual genomic loci at 500 bp bin resolution. Conservatively, more distant yet strong intrachromosomal contacts can be observed at 1 kb or 2.5 kb bin resolution. We show that chromatin internal to silent genomic regions has extensive, dense, and random internal contacts, while gene-rich, active chromatin forms “globules” ∼20–40 kb in length that are hierarchically packaged into “Regional Globule Clusters”—analogous to TADs. Further, we observe small euchromatic segments enriched with activating (H3K4me3 or H3K27ac) or repressive (H3K36me2) histone post-translational modifications that contact H3K9me3-marked constitutive heterochromatic regions, and many genes associating with silent chromatin are misregulated when heterochromatin is compromised (e.g. in a Δ*dim-5* strain), possibly reflecting a novel fungal gene regulation mechanism. All told, our high-resolution in situ Hi-C datasets of wild type *N.* *crassa* are valuable resources for the study of eukaryotic genome topology and should facilitate future studies in other conditions or genotypes to characterize fungal chromosome conformation.

## Materials and methods

### Strains, culture conditions, crosslinking, and isolation of *Neurospora spheroplasts*

Wild type (WT) *N.* *crassa* strains N150 and N3752 were used for all experiments; both strains are called “74-OR23-1VA” and share the same Fungal Genetics Stock Center number (FGSC#2489) but can be considered independent strains due to differing acquisition times and asexual laboratory propagation. Neurospora culture growth, formaldehyde crosslinking, and spheroplasting were performed essentially as described (Galazka *et al.* 2016; Klocko *et al.* 2016). The detailed Materials and Methods text describing the in situ Hi-C method as well as a comprehensive, step-by-step protocol for in situ Hi-C adapted to *N.* *crassa* are both provided in the Supplementary material.

### Hi-C library construction

Hi-C libraries were generally constructed as previously described (Lieberman-Aiden *et al.* 2009; Galazka *et al.* 2016; Klocko *et al.* 2016), but the protocol was adjusted to generate ligation products in the nucleus (in situ Hi-C) (Rao *et al.* 2014; Tanizawa *et al.* 2017), which more accurately reflects in vivo genomic contacts. The protocol was also refined for efficient use of reagents (Belaghzal *et al.* 2017). Specific changes include: spheroplast lysing by glass bead vortexing combined with permeabilizing nuclear membranes with SDS (Tanizawa *et al.* 2017); Klenow blunting of 5′ DNA overhangs in a smaller volume to maintain the required 30 μM nucleotide concentration but using less Biotin-14-dATP (Invitrogen cat# 19524-016); in situ ligating DNA strands in a smaller volume; and streamlining organic extraction of DNA ligation products.

### Preparation of Hi-C libraries for Illumina sequencing

Hi-C libraries for Illumina sequencing were prepared using a NEBNext Ultra II kit (New England Biolabs cat# E7645) with corresponding Multiplex Barcode sets (NEB cat# E7335 and E7500) according to the manufacturer’s protocol, with the following exceptions, as all steps were performed using the Hi-C library attached to the magnetic streptavidin beads: following the adapter ligation and USER enzyme digestion, magnetic beads were washed 5 times with 1x BW buffer [5 mM Tris-HCl (pH 8 at 25°C), 0.5 mM Na-EDTA, 1 M NaCl, 0.05% (v/v) Tween-20] and once with TE/10, and were resuspended in 15 μl TE/10 [10 mM Tris-HCl (pH 8 at 25°C), 0.1 mM sodium EDTA]; PCR enrichment of the barcoded Hi-C library off streptavidin beads used either 8 or 15 cycles (or an initial 15 PCR cycles, whereupon the beads were washed 3 times with TE/10, and were re-amplified by an additional 8 cycles, for a total of 23 cycles); and, following the separation of the aqueous PCR product from the magnetic beads, the PCR product was cleaned with a 1:1 ratio of Ampure XP beads (Agencourt, Beckman-Coulter) per the manufacturer’s protocol, resuspended in 25 μl TE/10, and quantified by a Qubit HS reaction. Prior to sequencing, all libraries were assessed for quality via Fragment Analyzer and quantity of barcoded DNA by qPCR [Genomics and Cell Characterization Core Facility (GC3F), University of Oregon]. Indexed in situ Hi-C libraries were pooled and sequenced on either an Illumina HiSeq 4,000 as 100 nucleotide (nt) paired-end sequencing runs or an Illumina NovaSeq 6000 as 59 nt paired-end sequencing runs at the Genomics and Cell Characterization Core Facility [GC3F] at the University of Oregon. Hi-C dataset fastq files are provided in Supplementary Table 1.

### Bioinformatic data analysis, including mapping of Hi-C libraries and merging of *Dpn*II and *Mse*I datasets

Paired-end reads were initially mapped to the corrected *Neurospora* genome assembly version 12 (nc12) (Galagan *et al.* 2003; Galazka *et al.* 2016). The *Mse*I dataset analysis allowed further correction of nc12 by providing locations for the unassigned Supercontigs 8–20; the new *N.* *crassa* genome assembly, here termed “version 14” (nc14), was used to construct all additional genome contact maps.

All in situ Hi-C contact maps and other analyses presented in this manuscript were generated using the program suite HiCExplorer (https://hicexplorer.readthedocs.io/en/latest/index.html; accessed 2022 September 3) (Ramírez *et al.* 2018), using version 3.5 for all analyses, except the program hicFindEnrichedContacts (version 2.2.3) for observed *vs.* expected matrix generation. Briefly, raw fastq files were mapped to the Neurospora genome (either nc12 or nc14) using bowtie2 (version 2.3), with the --local and --reorder flags, and the initial, high resolution (0.5 kb bin) Hi-C contact matrix was built with hicBuildMatrix using the default settings (double digest matrices were built using the restriction and dangling sequences of both *Dpn*II and *Mse*I); lower resolution matrices were created by the program hicMergeMatrixBins. In situ Hi-C matrix files are listed in Supplementary Table 2. Pearson correlation between replicates was determined and plotted by the command hicCorrelate, while the plots of Hi-C counts in relation to genomic distance for replicate matrices, or different merged dataset resolutions, were generated by the program hicPlotDistVisCounts. Comparison of matrices was performed with the program hicCompareMatrices. Images displaying bin contacts in the in situ Hi-C matrix were made with hicPlotMatrix, and Neurospora “TADs” were plotted with the commands hicFindTads and hicPlotTads. Contact maps are either presented as raw or Knight-Ruiz corrected (Knight and Ruiz 2013) images, the latter accounting for differences in restriction enzyme site distribution or sequencing bias of underlying DNA, while observed vs expected datasets are presented to show contact strength independent of genomic distance. Contact quantification was performed with the shell script process.sh, which uses the hicConvertFormat subprogram in hicExplorer to convert the Hierarchical Data Format version 5 [.h5] matrix produced from hicExplorer into a homer format matrix; the python script dataconvert.py converts the homer matrix into an NxN array, which is used by the python script epigenetic-mark-Quant-v2.py, adapted from Galazka *et al.* (2016), to calculate bins that are enriched for specific epigenetic marks and counts the number of enriched bins within and between chromosomes. All scripts are in the publicly available Klocko-Lab GitHub (https://github.com/Klocko-Lab/Chip_Quantification; accessed 2022 September 3).

As replicate Hi-C datasets are locally reproducible, *Dpn*II or *Mse*I replicate fastq files were independently merged, resulting in a combined *Dpn*II dataset with 269.9 million (M) reads (89.0M reads deemed “valid” by the stringent quality control standards used by HiCExplorer) and a merged *Mse*I dataset with 93.8M reads (20.4M valid reads). To generate a comprehensive dataset representative of the chromatin composition of the WT Neurospora genome from individual *Dpn*II (euchromatic-specific; GATC recognition sequence) and *Mse*I (heterochromatic-specific; TTAA recognition sequence) datasets, we calculated the percent of the Neurospora genome covered by H3K9me3 demarcating constitutive heterochromatin, which have an increased number of *Mse*I sites due to the AT-rich nature of the underlying DNA. To this end, we generated bed files of a previously published H3K9me3 merged dataset (Jamieson *et al.* 2016; Klocko *et al.* 2019), subtracted the end point from the starting point to calculate the number of basepairs covered by H3K9me3 at each region, summed the total number of bases, and divided the H3K9me3-covered bases by the genome size to get the percentage (15.76%) of the Neurospora genome covered by H3K9me3. The comprehensive in situ Hi-C dataset was built by combining raw *Dpn*II and *Mse*I fastq files that contain the appropriate numbers of valid *Mse*I or *Dpn*II PE100 reads that, when combined, would provide 15.76% *Mse*I-derived contacts and 84.24% *Dpn*II-derived contacts. Here, 21.4M reads (3.8M valid reads) were removed from *Mse*I replicate #4, meaning a fastq file containing 72.4M total *Mse*I reads (16.7M valid *Mse*I reads) was merged with a fastq file containing 270M total *Dpn*II reads (89.2M valid *Dpn*II reads). This single fastq dataset containing 342.2M *Dpn*II and *Mse*I reads (105.9M valid *Dpn*II and *Mse*I reads) was then used to build a high-resolution (0.5 kb bin) Hi-C contact matrix, and that comprehensive matrix (the “merged, single fastq” matrix) was used for subsequent contact analysis and generation of heatmaps for figures. A similar method was used to examine the robustness of merging *Dpn*II and *Mse*I datasets (Supplementary Fig. 15), where 23,340,020 valid reads from each of the 3 *Dpn*II replicate fastq files and 4,359,980 valid reads from each of the 3 *Mse*I replicate fastq files (giving 27.7M total valid reads at the percentages of the chromatin composition of the Neurospora genome) were selected and merged in every possible combination.

*Neurospora crassa* Chromatin Immunoprecipitation sequencing (ChIP-seq) datasets were reported previously (WT H3K9me3—merged from GSE68897 and GSE98911; WT H3K27me2/3—merged from GSE82222 and GSE100770; WT H3K27ac—GSE118495; WT CenH3—GSE71024; WT H3K4me3—GSE121356; WT/Ash1L (Δ*set-2*) H3K36me2—GSE118495) (Galazka *et al.* 2016; Jamieson *et al.* 2016; Klocko *et al.* 2016, 2019, 2020; Bicocca *et al.* 2018; Zhu *et al.* 2019). Datasets were downloaded and remapped to either the nc12 genome (Galazka *et al.* 2016) or our corrected nc14 genome. SAM file outputs were converted to sorted BAM files with SAMtools (Li *et al.* 2009), which were used to create bedgraph or bigwig files at 50 bp bin resolution with DeepTools (Ramírez *et al.* 2016) for display on Integrative Genomics Viewer (IGV) (Robinson *et al.* 2011); the count feature of the IGVtools program within IGV was also used to create TDF files at 50 bp bin resolution. IGV images of ChIP-seq enrichment tracks were used for figure construction.

Neurospora WT and Δ*dim-5* polyadenine messenger RNA sequencing (polyA mRNA-seq) datasets were previously reported (GSE82222) (Klocko *et al.* 2016). Here, processed HTseq count files (Anders *et al.* 2015) were downloaded and BED files of differentially expressed genes (here, gene expression in the mutant was up- or downregulated four times the WT expression level: increased genes have a log_2_ value of 2 or more and decreased genes are log_2_ = -2 or less) were created with DESeq2 (Love *et al.* 2014). IGV images of RNA-seq bed files were used for figure construction.

## Results

### In situ Hi-C of a wild type Neurospora strain using the restriction enzyme *Dpn*II primarily captures the chromosome conformation of euchromatic regions of the genome

Previous work elucidated the *N.* *crassa* genome organization at a lower resolution using proximity ligation Hi-C; the resulting contact matrices were generated and analyzed with a software pipeline that is no longer supported (Lieberman-Aiden *et al.* 2009; Galazka *et al.* 2016; Klocko *et al.* 2016). Prior to new Hi-C library construction, we chose to switch to HiCExplorer, a comprehensive and easy-to-use data analysis package (Ramírez *et al.* 2018). Re-analysis of the previously published proximity ligation Hi-C dataset of the wild type (WT) strain NMF39 (strain 74-OR23-1VA [FGSC #2489], asexually propagated in the Freitag lab, Oregon State University) (Galazka *et al.* 2016) confirmed the reproducibility of the HiCExplorer software package: a previously generated, yet unpublished, iteratively corrected (Imakaev *et al.* 2012) contact heatmap of the Neurospora LG II mirrored a Knight-Ruiz (Knight and Ruiz 2013) corrected contact heatmap generated by HiCExplorer (Supplementary Fig. 1), demonstrating the HiCExplorer package accurately generates and displays contact strength matrices for *N.* *crassa* Hi-C datasets.

**Fig. 1.**
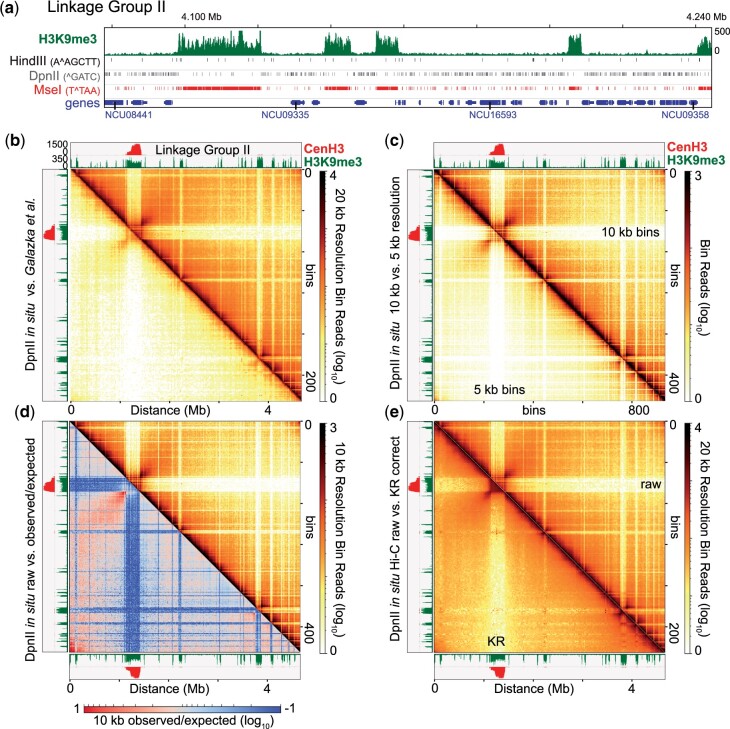
High-resolution chromosome conformation of *Neurospora crassa* using the restriction enzyme *Dpn*II. a) Integrative genomics viewer (IGV) (Robinson *et al.* 2011) image of a portion of Linkage Group II (LG II), showing tracks of H3K9me3 ChIP-seq (delineating heterochromatic regions), genes (track with rectangles; several NCU gene numbers are shown), and the restriction enzyme sites and recognition sequences of *Hind*III, *Dpn*II, and *Mse*I. Genomic distance indicated above the tracks, and ChIP-seq enrichment scale to the right. b) Heatmap of the chromosome conformation of LG II, generated from the merged *Dpn*II in situ Hi-C dataset, combining 4 replicates (this work; above diagonal), and the previously published proximal ligation Hi-C (*Hind*III) dataset (below diagonal) (Galazka *et al.* 2016). Each heatmap is displayed as the raw read count per 20 kilobase (kb) bins; here, and in images throughout this work, only one half of an individual square plot is included in figures to allow comparisons, since each contact heatmap generated is a mirror image reflected about the diagonal. All images in this manuscript are similarly displayed, with the number of bins (vertical markings) and genomic distance, in megabases (Mb) (horizontal markings) shown on the plot axes. Scale bars, provided on all images or groups of images, indicate the number of mapped reads per bin, either on a log_10_ scale (log transformed) or as absolute values. CenH3 and H3K9me3 ChIP-seq tracks presented above and to the left to indicate the centromeric and heterochromatic regions of LG II, respectively; ChIP-seq enrichment scales shown; similar scales are used for ChIP-seq enrichment tracks throughout the manuscript. c) Heatmap displaying the merged *Dpn*II in situ Hi-C dataset plotting raw read counts per 10 kb bin (above diagonal) or 5 kb bin (below diagonal). d) Heatmap displaying the merged *Dpn*II in situ Hi-C dataset of raw read counts (above diagonal) and the calculated observed *vs.* expected plot (below diagonal) to highlight genomic interactions different from those expected, based on the inverse relationship of contact strength *vs.* linear distance. Both plots are at 10 kb resolution; scale bar of log_10_ observed *vs.* expected contact strength shown below. e) Heatmap displaying the merged *Dpn*II in situ Hi-C dataset of raw read count per 20 kb bin (above diagonal) and the Knight-Ruiz (KR) corrected (below diagonal) read count per 20 kb bin.

The published proximity ligation Hi-C dataset captured chromatin interactions using the restriction enzyme *Hind*III (Galazka *et al.* 2016), which cleaves a less common restriction site sequence (A^AGCTT; sites every 4,096 basepairs [bp] on average) (Fig. 1a). However, recent work has cast doubt on the accuracy of proximity ligation Hi-C (Rao *et al.* 2014; Nagano *et al.* 2015) and we were concerned that the paucity of *Hind*III restriction sites would fail to capture crucial long-range contacts at individual genes. Therefore, we adapted and refined our Hi-C protocol to capture chromosome conformation in fungal nuclei (in situ) (Rao *et al.* 2014; Tanizawa *et al.* 2017) using *Dpn*II (^GATC; sites every 256 bp on average; enzymatic activity not inhibited by cytosine methylation, allowing ligation of methylated genomic regions), as *Dpn*II restriction sites are more abundant in gene-rich euchromatin (Fig. 1a). Using a single WT strain (N150; 74-OR23-1VA), we initially generated 3 replicate *Dpn*II in situ Hi-C libraries amplified with 15 PCR cycles for the final library barcoding step (Supplementary Fig. 2a), akin to previous Hi-C library generation (Galazka *et al.* 2016; Klocko *et al.* 2016); these replicate datasets are highly similar when compared (Supplementary Fig. 2a). However, Neurospora heterochromatic regions, rich in adenine and thymine DNA basepairs, are known to be depleted by increased numbers of PCR cycles during library barcoding (Ji *et al.* 2014), suggesting 15 PCR cycle libraries may not accurately report heterochromatic contacts. Therefore, we repeated the Hi-C library construction using the same frozen spheroplast samples of WT N150, amplifying the final Hi-C library with only 8 PCR cycles (Ji *et al.* 2014); we also reamplified our Hi-C ligation products bound to streptavidin beads (previously with 15 PCR cycles) with another 8 cycles (a “23 PCR cycle library”) to assess if library reamplification also underrepresented AT-rich regions. Comparison of the 8 and 15 PCR replicates show heterochromatin depletion in the latter (Supplementary Fig. 2b), while the “23 PCR cycle” replicates (Supplementary Fig. 2c) qualitatively exhibit even greater AT-rich depletion than 15 PCR cycle libraries (Supplementary Fig. 2d). Unsurprisingly, independently merging the 8, 15, and 23 PCR cycle replicates into separate datasets for comparison shows similar results: the 8 PCR cycle merged matrix has more AT-rich heterochromatin signal as compared to the merged 15 and 23 PCR cycle datasets (Supplementary Fig. 3). Given the depletion of AT-rich DNA, we abandoned the 15 and 23 PCR cycle in situ Hi-C datasets, and from this point forward, exclusively analyzed the 8 PCR cycle *Dpn*II in situ Hi-C libraries.

To confirm our 3 N150 replicates with a biological replicate of an independently propagated strain, we generated another *Dpn*II in situ Hi-C library of the WT strain N3752 (74-OR23-1VA) for 4 WT *Dpn*II chromosome conformation replicates. Heatmaps of contact matrices indicate the 4 replicates are highly reproducible and include strong contacts off-diagonal at the highest resolutions (Supplementary Fig. 4a). When the replicate contact matrices were compared, 1 replicate of N150 and the N3752 replicate show fewer euchromatic-heterochromatic contacts (Supplementary Fig. 4b), possibly reflecting subtle differences in the distribution of nuclei in cell cycle stages (Naumova *et al.* 2013; Tanizawa *et al.* 2017). Raw and corrected replicates correlate well: each display the inverse relationship between contact strength and genomic distance typical of Hi-C datasets (Supplementary Fig. 4, c and d), and Pearson scores of replicate Hi-C contact matrices range between 0.86 and 0.92 (Supplementary Fig. 4e), verifying replicate robustness. We note that all in situ Hi-C contact matrices in this manuscript are derived from the average genome topology across an entire population of unsynchronized nuclei, which limits the detail of chromatin structure obtained from individual nuclei.

As local euchromatic contacts were highly reproducible, we merged all 4 replicates into a single *Dpn*II in situ Hi-C dataset, which when combined, had 269.9M total reads, 89.0M of which were deemed valid Hi-C ligation products using the stringent quality control standards in HiCExplorer; considering the 136,425 *Dpn*II sites in the Neurospora genome, this reflects ∼654 contacts per site on average. To assess the accuracy of the in situ protocol at capturing chromatin contacts exclusively in the nucleus, we examined the number of ligation products containing reads that map to the mitochondrial genome. Our merged *Dpn*II in situ Hi-C data have substantially reduced numbers of mitochondrial-specific reads as Hi-C ligation products relative to the published proximity ligation dataset (Galazka *et al.* 2016; Supplementary Fig. 5). These data suggest our merged *Dpn*II in situ Hi-C dataset accurately captured genomic contacts in the nucleus.

To assess if this new dataset reflects Neurospora chromosome conformation at a high resolution, we compared the merged in situ *Dpn*II dataset to the published proximity ligation *Hind*III data containing ∼11.2M valid contacts (Galazka *et al.* 2016); the latter is typically displayed with chromosome-level contact matrices using 40 kb bins. Our merged in situ raw contact count matrix displays a greater saturation of data points and density of information across a single, representative chromosome (LG II) at a higher (20 kb) resolution (Fig. 1b), reflecting the capture of a high number of short- and long-range contacts in euchromatic regions. Heterochromatin, marked by H3K9me3 and/or the centromeric histone variant CenH3 (Tamaru *et al.* 2003; Smith *et al.* 2011), has few contacts with euchromatin (Fig. 1b; above diagonal), consistent with the compartmentalization of silent and active chromatin (Lieberman-Aiden *et al.* 2009; Rao *et al.* 2014). The other 6 individual Neurospora chromosomes (Supplementary Fig. 6) and the entire genome (Supplementary Fig. 7) show similar results; intrachromosomal contacts are slightly enriched relative to contacts between chromosomes across the whole genome. At higher resolutions, including 10 and 5 kb bins, numerous local intrachromosomal contacts are observed (Fig. 1c), demonstrating the depth of our merged *Dpn*II dataset. To examine chromosomal interactions independent of genomic distance, we plotted the log_10_ change in observed contacts within the merged *Dpn*II dataset relative to the expected inverse relationship between contact strength and genomic distance (e.g. the contact strength between 2 loci should decrease as the distance separating the loci increase); any deviations between observed contact numbers and the number of expected interactions highlight strong interactions, or a dearth of contacts, between genome features not occurring by chance. Observed contacts are massively increased above expected at centromere flanks and subtelomeres, while few contacts between heterochromatin and euchromatin are observed (Fig. 1d), consistent with heterochromatic regions strongly associating and thereby segregating euchromatin across the Neurospora genome (Galazka *et al.* 2016; Klocko *et al.* 2016). strong

To balance our merged matrix, we employed Knight-Ruiz (KR) correction to account for differences in restriction site number or potential ligation/sequencing bias in our Hi-C libraries (Knight and Ruiz 2013; Ramírez *et al.* 2018), possibly at the expense of local 3-dimensional chromatin structures, as correcting bin signals has the potential to nullify chromatin contacts; to allow for objective data interpretation, many figures in this manuscript present both raw and KR corrected matrix heatmaps. Compared to the raw contact matrix, the KR-corrected *Dpn*II dataset displays uniform intrachromosomal euchromatic contacts, while more distant heterochromatic region interaction signals became stronger (Fig. 1e, Supplementary Fig. 6 and 7; below diagonal). We note some smaller heterochromatic regions have increased euchromatic contacts upon KR-correction (evident by the loss of low-signal horizontal/vertical lines emanating from heterochromatic regions), suggesting matrix correction may somehow bias chromatin contacts between compartments. All told, our merged in situ *Dpn*II Hi-C dataset captures the Neurospora chromosome conformation at a high resolution.

### Heterochromatin specific chromosome conformation is revealed by in situ Hi-C with *Mse*I

While our *Dpn*II in situ Hi-C dataset efficiently monitors euchromatic contacts, we were concerned by the paucity of silent chromatin interactions in these data. Previous reports demonstrated strong interactions in heterochromatic regions in Neurospora (Galazka *et al.* 2016; Klocko *et al.* 2016), but the reduced number of *Dpn*II sites in AT-rich DNA may compromise any assessment of long-range contacts for heterochromatic DNA (Fig. 1a). Indeed, heatmaps of raw contact numbers display few interheterochromatic contacts, but KR-corrected heatmaps, which account for restriction site differences in interaction bins, show strong, long-range association of heterochromatic regions (Fig. 1e). Therefore, we adapted our Hi-C protocol for the restriction enzyme *Mse*I, as its T^TAA recognition sequence is highly prevalent in AT-rich, heterochromatic DNA (Fig. 1a;  Klocko *et al.* 2019), allowing efficient capture of chromosome conformation in gene-poor, H3K9me3-marked silent regions of the Neurospora genome. We performed in situ Hi-C using *Mse*I, generating 2 replicates of the WT N150 strain and a third biological replicate of the independently propagated WT strain N3752. Heatmaps of *Mse*I replicates show reproducibility in heterochromatic contact capture across a single chromosome, with strong Hi-C signal presenting at heterochromatic regions in high-resolution matrices (Supplementary Fig. 8a). The 3 replicates show similar heterochromatic interactions with variability in medium range off-diagonal euchromatic contacts (within ∼100 kb), possibly due to variable capture across the reduced number of *Mse*I sites found in euchromatin (Fig. 1a, Supplementary Fig. 8b). Each *Mse*I replicate dataset is well correlated, with similar contacts and strong Pearson correlation scores (Supplementary Fig. 8c). All raw and KR-corrected replicate matrices display the typical, inverse relationship between contact strength and genomic distance in plots of genomic distance versus Hi-C contact numbers (Supplementary Fig. 8, d and e).

Since the 3 *Mse*I replicates are reproducible, we merged the replicates into a single *Mse*I in situ Hi-C dataset, containing 72.4 million reads, 16.7 million of which were deemed valid by HiCExplorer quality control parameters. Similar to *Dpn*II Hi-C datasets, *Mse*I Hi-C datasets show few mitochondrial ligation products, confirming the accuracy of the in situ Hi-C protocol (Supplementary Fig. 5). Considering the 195,726 *Mse*I sites across the Neurospora genome, the number of *Mse*I reads represents ∼85 contacts per site—fewer than our *Dpn*II data—but given the density of *Mse*I sites in heterochromatic AT-rich DNA (Fig. 1a), these ligation products are more likely to capture internal folding of, and contacts between, silent chromatin. In support, plots of genomic distance *vs.* Hi-C read counts of merged matrices (both raw and KR-corrected) show increased short-range and reduced long-range contacts, presumably within and between heterochromatic regions (below) in *Mse*I contact matrices relative to *Dpn*II datasets (Supplementary Fig. 9, a and b).

**Fig. 2.**
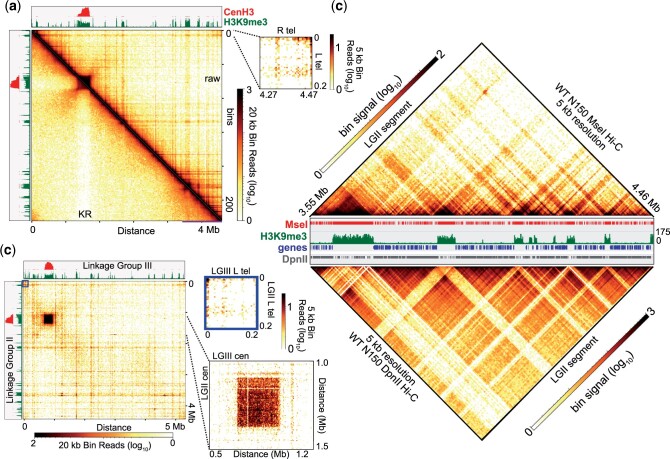
High-resolution Hi-C of *Neurospora crassa* using the restriction enzyme *Mse*I. a) Heatmap displaying the merged *Mse*I in situ Hi-C dataset of LG II, either plotting raw read counts (above diagonal) or KR-corrected counts (below diagonal), at 20 kb bins. The magnified heatmap shows the intrachromosomal interaction between the left and right telomeres of LG II at 5 kb resolution. CenH3 (centromeric) and H3K9me3 (heterochromatic) ChIP-seq enrichment tracks presented above and to the left of each plot. Horizontal line at bottom of heatmap highlights region displayed in panel (c). b). Heatmap displaying the interactions between LG II and LG III in the merged *Mse*I in situ Hi-C dataset at 20 kb. Magnified plots at 5 kb resolution show the interactions between the LG II/LG III centromeres (dashed lines) or the interactions between the left subtelomeres of LG II and LG III (box). c). Heatmaps of *Mse*I (top) and *Dpn*II (bottom) contacts at 5 kb bin resolution of the right arm of LG II (highlighted in A by horizontal line). Both triangle heatmaps were cropped and rotated 45° from square (reflective) heatmaps. Central IGV image displays tracks of *Mse*I and *Dpn*II restriction sites, H3K9me3 ChIP-seq enrichment and genes.

Raw contact count heatmaps of the merged *Mse*I in situ Hi-C matrix across LG II mainly display increased Hi-C interactions within and between constitutive heterochromatic regions: internally, strong contacts occur across the entire length of silent regions, while strong, long-range contacts involve distant heterochromatic regions across Megabases (Mb) of linear chromosomal distance; strong euchromatic contacts are primarily restricted to neighboring bins on-diagonal (Fig. 2a; above diagonal). Similar results were observed for the other 6 chromosomes (Supplementary Fig. 10, above diagonal). KR correction of *Mse*I matrices eliminates most long-range heterochromatic contacts, potentially due to disparate *Mse*I site positions and resultant discrepancies in silent and active chromatin contact strength (Fig. 2a, Supplementary Fig. 10; below diagonal), again highlighting our caution in exclusively relying on KR-corrected matrices to elucidate valid chromatin structures. Our *Mse*I data effectively captures intra- and interchromosomal heterochromatic interactions, as strong contacts between centromeres are observed, as are interactions between subtelomeres; interspersed heterochromatic regions also associate, but these silent regions are segregated from neighboring euchromatin, as shown in single chromosome (Fig. 2, a and b, Supplementary Fig. 10) and whole genome contact heatmaps (Supplementary Fig. 11), further supporting the bundling of heterochromatin (Galazka *et al.* 2016).

To directly compare the heterochromatic contacts captured in *Mse*I in situ Hi-C matrices to our euchromatic-specific *Dpn*II in situ Hi-C data, we examined the *Dpn*II and *Mse*I datasets on a magnified, ∼1 Mb region of the right arm of LG II that extends from ∼3.5 to 4.5 Mb and encompasses several heterochromatic regions. The *Mse*I Hi-C data effectively captured heterochromatin contacts in this region, which are displayed as strong “triangles” of Hi-C interactions (Fig. 2c, top). Adjacent H3K9me3-marked regions are more apt to associate, but strong contacts between heterochromatic regions separated by distances of ∼1 Mb were still observed (Fig. 2c, top). In contrast, the strongest contacts in gene-rich euchromatin occur locally, and despite the proximity of silent chromatin on the linear chromosome, few euchromatin-heterochromatin interactions are seen at this resolution (Fig. 2c; bottom). In fact, neighboring euchromatic regions are more inclined to associate (Fig. 2c; bottom). Taken together, *Dpn*II and *Mse*I in situ Hi-C have the capacity to independently capture and assess euchromatic and heterochromatic contacts, respectively, across the Neurospora genome.

### *Mse*I in situ Hi-C facilitates the correction of the *N. crassa* reference genome

Initially, we used version 12 of the Neurospora genome (nc12) for our Hi-C data processing; this genome version contains 7 chromosomes (Linkage Groups [LGs]) and 13 other Supercontigs (SCs), numbered 8–20, whose location in the genome could not be determined, possibly due to repetitive sequences at SC boundaries (Galagan *et al.* 2003; Galazka *et al.* 2016). Our *Mse*I data show strong interactions between the unplaced SCs and LGs I and V, the latter 2 exhibiting gaps in local contacts (Supplementary Figs. 12a and 13a), suggesting SCs 8–20 are found in these chromosomes. Therefore, we corrected the Neurospora reference genome, creating version 14 (nc14; the number was chosen to account for a previous correction of LG VI) (Galazka *et al.* 2016) by placing the DNA sequences of SCs 8–20 in the reference genome fasta file using *Mse*I Hi-C data to guide sequence placement. Despite these improvements, nc14 still contains numerous gaps that could not be corrected with Hi-C data.

First, we noticed that SCs 10, 11, 13, and 14 strongly associate with a small heterochromatic region on LG I, centered ∼6.40 Mb in the nc12 genome (Supplementary Fig. 12, a and b). Comparison of these small SCs showed their approximate order: the strongest contacts between these small SCs highlight their order in the LG (Supplementary Fig. 12c), while binary comparisons between the small SCs and LG I (from 6.15 to 6.55 Mb) confirmed insertion endpoints (Supplementary Fig. 12d). Together, these data suggest an order of SC 14, 10, 13, with SC 11 slightly downstream, all of which are internal to the LG I heterochromatic region (Supplementary Fig. 11b). Further, we noticed SC 17 is located at ∼3.7 Mb in LG I, within the centromere, as shown by the binary comparison of SC 17 and the LG I centromere (Supplementary Fig. 12e). We corrected LG I in the nc14 genome by placing SCs 14, 10, 13, 11, and 17 into locations with unknown “N” bases, which creates a ∼200 kb larger heterochromatic region from ∼6.4 to 6.65 Mb and extends the centromere (Supplementary Fig. 12b). Remapping either our *Mse*I Hi-C reads or published H3K9me3 Chromatin Immunoprecipitation sequencing (ChIP-seq) data (Jamieson *et al.* 2016; Klocko *et al.* 2019) to the amended nc14 genome accurately reflects internal and long-range heterochromatic contacts across LG I and H3K9me3 enrichment of the corrected silent regions (Supplementary Fig. 12, b and f).

The left arm of Neurospora LG V was also corrected: this is the location of the repeated ribosomal DNA gene copies comprising the Nucleolus Organizing Region (NOR). Previous work suggested SCs 8, 9, 12, 15, 16, 18, and 19 localize to LG V, but the order was mostly unknown (Perkins *et al.* 2001). Our *Mse*I Hi-C data independently confirmed that these smaller SCs are present on LG V (Supplementary Fig. 13a), and examination of the SC 9 sequence showed the presence of telomeric repeats, supporting the hypothesis that SC 9 terminates the LG V left arm (Supplementary Fig. 13b) (Perkins *et al.* 2001). Interchromosomal comparisons of smaller SCs confirmed that the LG V left arm begins with SC 9, followed by SCs 8, 15, 19, 18, 12, and 16 (Supplementary Fig. 13, c and d). We inserted these SCs into the nc14 genome for improving the LG V left arm sequence and remapped our *Mse*I data. The resulting Hi-C heatmap clarifies the organization of LG V, showing that the NOR on the left arm of LG V is sequestered from most other genomic loci, although some heterochromatic contacts exist (Supplementary Fig. 13e). Closer examination of the *Mse*I Hi-C data of the LG V left arm shows a cross-shaped pattern centered on the rDNA gene NCU15719 (Supplementary Fig. 13f), which was also observed on SC 8 in nc12 (Supplementary Fig. 13b), suggesting the sequence of this rDNA gene is repeated multiple times, preventing discrimination between individual rDNA gene copies in genomic experiments (note: SC 20 is identical to NCU15719 and was excluded). While Hi-C contact data implies promiscuity in NCU15719 genomic contacts, the increased copy number of this gene may bias the Hi-C data, making it incorrectly appear that the NCU15719 rDNA gene has increased Hi-C interactions. Previous estimates place the total number of rDNA copies between 130 and 172 (Butler and Metzenberg 1989), and since older versions of the Neurospora genome had 55 rDNA gene copies (Perkins *et al.* 2001; Galagan *et al.* 2003), NCU15719 could be repeated ∼75 to 117 times, a copy number supported by the enrichment of CenH3 ChIP-seq data relative to background (Supplementary Fig. 13f, legend). All told, our *Mse*I Hi-C data assisted in creating a more accurate reference genome for *N.* *crassa*.

### The complete genome topology of *N. crassa* is revealed upon merging *Dpn*II and *Mse*I datasets at proportions consistent with the chromatin composition of the Neurospora genome

Using *Dpn*II and *Mse*I restriction enzymes, we independently captured the chromosome conformation of GC-rich euchromatic and AT-rich heterochromatic regions, respectively (above). To more comprehensively explore *Neurospora’s* genome topology, we envisioned merging fastq files of our *Dpn*II and *Mse*I datasets and building a single contact matrix. First, to test if merging *Dpn*II and *Mse*I fastq files into a single matrix would reproducibly and accurately present Hi-C contact data, we built 9 different fastq file datasets to examine if unique combinations of Hi-C reads produce similar contact matrices. To this end, we selected valid reads from 3 *Dpn*II replicates and 3 *Mse*I replicates at a ratio representative of the chromatin composition of the Neurospora genome: each *Dpn*II replicate fastq file had 23.34M (84.24%) valid reads and each *Mse*I replicate fastq file had 4.36M (15.76%) valid reads; the latter mirrors the percentage of genomic H3K9me3 (see *Materials and Methods*). Upon merging all combinations of *Dpn*II and *Mse*I replicate fastq files, contact matrices were built from the 9 fastq datasets, each with 27.7M valid reads, and Hi-C contacts were displayed in heatmaps. The resulting 9 heatmaps are essentially identical (Supplementary Fig. 15a), and binary comparisons of Hi-C contacts in the 9 matrices are highly correlated, with Pearson correlation values between 0.83 and 0.99 (Supplementary Fig. 15b). These data, along with the similarity to a double digest dataset (below), demonstrates that merging fastq files to produce a single in situ Hi-C contact matrix is a valid approach.

**Fig. 3.**
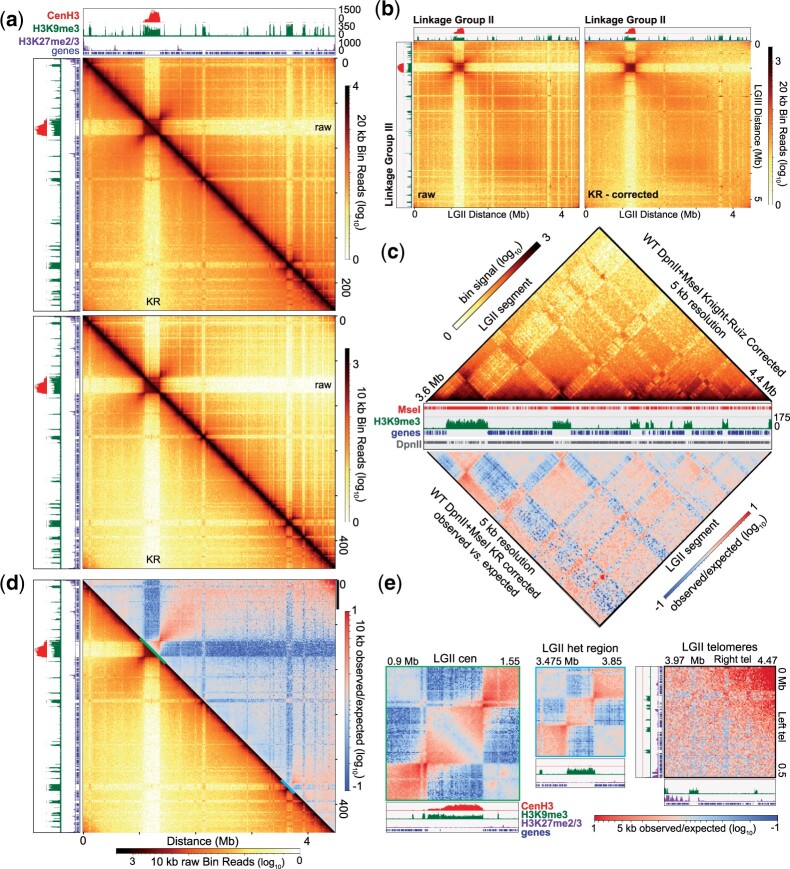
Comprehensive genome organization of *Neurospora crassa* created from merging *Dpn*II (euchromatic) and *Mse*I (heterochromatic) fastq files to build a single contact matrix. a). Heatmaps displaying the raw (above diagonal) and Knight-Ruiz (KR) corrected read counts (below diagonal) of genomic interactions at 20 kb (top heatmap) and 10 kb (bottom heatmap) bin resolution of the complete in situ Hi-C dataset, created from merging *Dpn*II and *Mse*I fastq files into a single fastq file, which was processed into a single contact matrix to avoid mathematical bias; this single matrix contains valid DpnII (89.24M) and MseI (16.69M) Hi-C reads at a ratio that accurately depicts the percentages of euchromatin (84.24%) and heterochromatin (15.76%), respectively, in the Neurospora genome (see *Materials and Methods*). CenH3, H3K9me3, and H3K27me2/3 ChIP-seq enrichment tracks, as well as a gene track, presented above and to the left of each plot. b). Heatmaps plotting interchromosomal contacts at 20 kb bin resolution between LG II and LG III created using raw read count (left) or the KR-corrected read count (right) matrices. CenH3 and H3K9me3 ChIP-seq enrichment tracks presented above and to the left of each plot. c). Triangle heatmaps of KR-corrected read count (top) and the plot of the calculated observed vs expected of KR-corrected read count of Hi-C contacts at 5 kb bin resolution of the right arm of LG II (identical to the region shown in Fig. 2c). Both triangle heatmaps were cropped and rotated 45° from square (reflective) heatmaps. Central IGV image displays tracks of *Mse*I (TTAA) and *Dpn*II (GATC) restriction sites, H3K9me3 ChIP-seq enrichment and genes. d). Heatmap of the calculated observed *vs.* expected signal of KR-corrected read count (above diagonal) and the KR-corrected read counts of the complete *Dpn*II-*Mse*I in situ Hi-C dataset (below diagonal) at 10 kb bin resolution. Centromeric, intratelomeric, and interspersed heterochromatic regions highlighted in E are marked by colored lines. e) Heatmaps of the calculated observed vs expected KR-corrected contacts for the centromere, an interspersed heterochromatic region , and intrachromosomal subtelomere interactions of LG II highlighted in panel (d). CenH3, H3K9me3, and H3K27me2/3 ChIP-seq enrichment, as well as gene tracks presented below each plot.

Next, we generated a comprehensive *Dpn*II and *Mse*I Hi-C dataset of the Neurospora genome by merging fastq files with percentages of valid Hi-C reads that reflect the Neurospora genome chromatin, mapping those reads to the nc14 reference genome, and subsequently building the contact matrix; we note that merging preprocessed matrices introduces mathematical bias (Supplementary Fig. 14). This comprehensive dataset has 342M reads, 106M of which are considered valid Hi-C contacts (Table S1). Plots of genomic distance *vs.* raw and KR-corrected contact numbers across multiple resolutions, from 50 to 1 kb bins, showed the typical inverse relationship in the number of Hi-C contacts relative to genomic distance for this single fastq contact matrix. Even at the highest resolutions, bins separated by ∼200 kb to 1 Mb of linear genomic distance average at least 1 contact, indicating high contact data depth (Supplementary Fig. 9, c and d). Raw and KR-corrected heatmaps of this comprehensive *Dpn*II/*Mse*I merged, single fastq dataset at 20 and 10 kb bin resolution across LG II show similar interactions to the *Dpn*II-only heatmap, but heterochromatic contacts are more prevalent (compare Figs. 3a and 1e). In particular, the KR-corrected matrix displays higher saturation of, and more detail for, silent chromatin contacts, including intracentromeric and long-range heterochromatic region interactions (Fig. 3a); the other 6 Neurospora chromosomes have similarly improved heterochromatin contacts (Supplementary Fig. 16). Long-range interactions between chromosomes are readily observed in raw and KR-corrected contact matrices, including strong bundling of heterochromatin and contacts between euchromatic chromosome arms (Fig. 3b). Across the entire genome, the 7 centromeres associate but are isolated from other chromosomal features, including the 14 independently clustered telomeres. Subtle contacts between the euchromatic arms of the 7 Neurospora chromosomes are also prevalent (Supplementary Fig. 17). The right arm of the KR-corrected LG II at a high resolution (5 kb bins) elucidates topological differences between silent and active chromatin (Fig. 3c), as heterochromatin strongly associates across hundreds of kilobases of linear distance, while regions of gene-rich active DNA are more apt to contact nearby euchromatin and remain segregated from heterochromatin.

To examine chromatin interactions independent of genomic distance, we plotted the difference in the observed Hi-C contact signal relative to the expected signal—derived from the inverse relationship of contact strength to genomic distance—across and internal to a single chromosome (LG II) (Figs. 3c, bottom, d and e), all 7 Neurospora chromosomes (Supplementary Fig. 18), and across the entire Neurospora genome (Supplementary Fig. 19). We observe centromeres and interspersed heterochromatic regions, as well as the flanking euchromatic DNA surrounding these silent regions, contact more than expected, indicating that internal compaction of heterochromatin causes neighboring euchromatin to strongly interact (Fig. 3, d and e, Supplementary Fig. 18); centromere clustering across the genome is also observed (Supplementary Fig. 19). Similarly, intra- and interchromosomal subtelomeres show strong observed contacts (Fig. 3, d and e, Supplementary Figs. 18 and 19), indicating chromosome folding facilitates the association of subtelomeres. Closer examination of Hi-C contacts independent of genomic distance on the right arm of LG II shows strong interactions between heterochromatic regions, increased associations between euchromatic regions, and few interactions between silent and active chromatin (Fig. 3c), although the latter intercompartment contacts are possibly depleted in this dataset, given that merging datasets with different underlying biases impacts matrix balancing (see Discussion). Despite this caveat, our comprehensive in situ Hi-C dataset, which merges independent *Dpn*II and *Mse*I datasets as a single fastq for generating the contact matrix, collectively illuminates the interactions of individual genome features at a high resolution.

### Double digest in situ Hi-C of *N. crassa* elucidates fungal genome topology and supports merging *Dpn*II and *Mse*I fastq files for a comprehensive Hi-C dataset

To expand our analysis of Neurospora genome architecture and confirm the robustness of our merged *Dpn*II and *MseI* dataset, we generated *Dpn*II/*Mse*I double digest in situ Hi-C datasets, as ligation of DNA between euchromatic and heterochromatic regions may be impacted in single enzyme Hi-C. Since blunting of sticky ends occurs before ligation in Hi-C, differentially digested DNA molecules can still be ligated (Lafontaine *et al.* 2021). We generated 5 double digest replicates of the N150 strain and a biological replicate using the N3752 strain. While our double digest replicates have varying valid read numbers (Supplementary Table 1), all display similar Hi-C contacts at a low resolution across a single chromosome (LG II) and at a high resolution across a small portion of the LG II right arm (Supplementary Fig. 20a); 2 replicates have mostly on-diagonal contacts, possibly due to reduced valid read numbers (Supplementary Table 1). All replicates, both as raw and KR corrected 50 kb bins, display similar inverse relationships between genomic distance and Hi-C contact numbers (Supplementary Fig. 20, b and c), and have high Pearson correlation scores when double digest replicates were compared in pairs (Supplementary Fig. 20d).

**Fig. 4.**
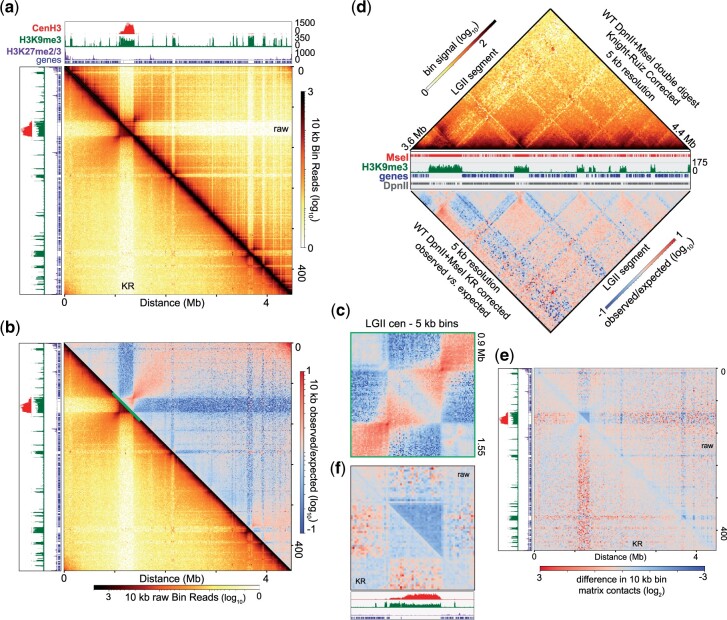
Comprehensive genome organization of *Neurospora crassa* derived from the *Dpn*II and *Mse*I double digest contact matrices. a) Heatmap displaying the raw (above diagonal) and Knight-Ruiz (KR) corrected read counts (below diagonal) of genomic interactions 10 kb bin resolution of the DpnII and MseI double digest in situ Hi-C dataset, which contained 76.5M valid reads. CenH3, H3K9me3, and H3K27me2/3 ChIP-seq enrichment tracks, as well as a gene track, presented above and to the left of the heatmap. b) Heatmap of the calculated observed vs expected signal of KR-corrected read count (above diagonal) and the KR-corrected read counts of the *Dpn*II and *Mse*I double digest in situ Hi-C dataset (below diagonal) at 10 kb resolution. Centromeric region highlighted in C is marked by a diagonal line. c) Heatmap of the calculated observed vs expected signal of the KR-corrected read counts across the centromere at 5 kb resolution. d) Triangle heatmaps of KR-corrected read count (top) and the plot of the calculated observed vs expected signal of KR-corrected read count at 5 kb bin resolution of the right arm of LG II, as in Fig. 3c, e, f) Heatmaps displaying the log_2_ ratio of in situ Hi-C contacts at 10 kb resolution comparing the 76.5M valid reads of the double digest to a merged fastq file that combines fastq files containing 64.5M valid DpnII reads with 12.0M valid MseI reads (76.5M valid reads overall), processed as a single matrix. The change in raw read counts (above diagonal) or KR-corrected counts (below diagonal), across (e) LG II or (f), the centromere of LG II is displayed. CenH3, H3K9me3, and H3K27me2/3 ChIP-seq enrichment, as well as gene tracks presented with each heatmap.

Given the similarity of double digest replicates, we merged all replicates into a single *Dpn*II/*Mse*I double digest dataset that comprises 456M reads, 76.5M of which are valid Hi-C contacts (Supplementary Table 1)—less than our merged, single fastq dataset (above) but vastly more than the published proximity ligation dataset (Galazka *et al.* 2016). Graphs of raw or KR corrected Hi-C contacts in the double digest vs genomic distance at multiple resolutions show the typical inverse relationship between Hi-C contacts and genomic distance (Supplementary Fig. 9, e and f). Heatmaps of raw and KR-corrected interactions of the merged double digest dataset across LG II at 10 kb bin resolution are highly comparable to heatmaps of the merged, single fastq dataset, with the exception that long-range heterochromatic interactions are less prevalent (compare Figs. 4a and 3a). Similar contacts are observed for the other 6 Neurospora chromosomes and genome wide (Supplementary Figs. 21 and 22). Plots of the observed *vs.* expected double digest dataset interactions, which highlight nonrandom contacts independent of genomic distance, show a similar chromatin topology as the merged, single fastq dataset: reduced heterochromatin-euchromatin interactions, but increased telomere bundling and a greater propensity of centromere flanks to contact (Fig. 4b), although the double digest dataset displays slightly less—but still overall increased—compaction within centromeres (Fig. 4c; compare to Fig. 3e). Closer examination of the LG II right arm shows strong contacts between euchromatic regions despite the presence of intervening heterochromatin (Fig. 4d, top); increased association of euchromatic regions is highlighted in plots of contacts independent of genomic distance (Fig. 4d, bottom). We observe reduced long-range heterochromatic contacts across the LG II right arm (Fig. 4d), possibly due to the increased likelihood of forming ligation products between silent and active DNA in the double digest reaction.

To compare the double digest dataset to the single fastq merged dataset, we merged 64.5M valid DpnII reads with 12.0M valid MseI reads (76.5M valid reads overall) in a single fastq file, mapped the reads to nc14, built the contact matrix, and compared this merged dataset to the double digest dataset across and within LG II to highlight any discrepancies between these 2 Hi-C datasets. While euchromatic contacts are virtually identical in raw and KR-corrected double digest datasets relative to the merged, single fastq Hi-C dataset (Fig. 4, e and f), the raw Hi-C contact matrix of the double digest dataset has reduced contacts within and between heterochromatic regions (Fig. 4, e and f, top). KR-correction of the double digest dataset partially alleviates the paucity of heterochromatic contacts but increases heterochromatic-euchromatic interactions (Fig. 4, e and f, bottom). Similar results are observed across all chromosomes and throughout the entire Neurospora genome (Supplementary Figs. 23 and 24a). Quantification of strong interactions between H3K9me3-marked regions independent of genomic distance (e.g. in observed vs expected matrices) shows reduced numbers of heterochromatic contacts in the double digest dataset, particularly for contacts between interchromosomal heterochromatic regions (Supplementary Fig. 24, b and c). Despite this subtle reduction in heterochromatic contacts, we conclude that our *Dpn*II/*Mse*I double digest dataset effectively captures Neurospora genome topology in a manner similar to the merged, single fastq *Dpn*II and *Mse*I contact matrix.

### Examination of chromatin folding at a high resolution

**Fig. 5.**
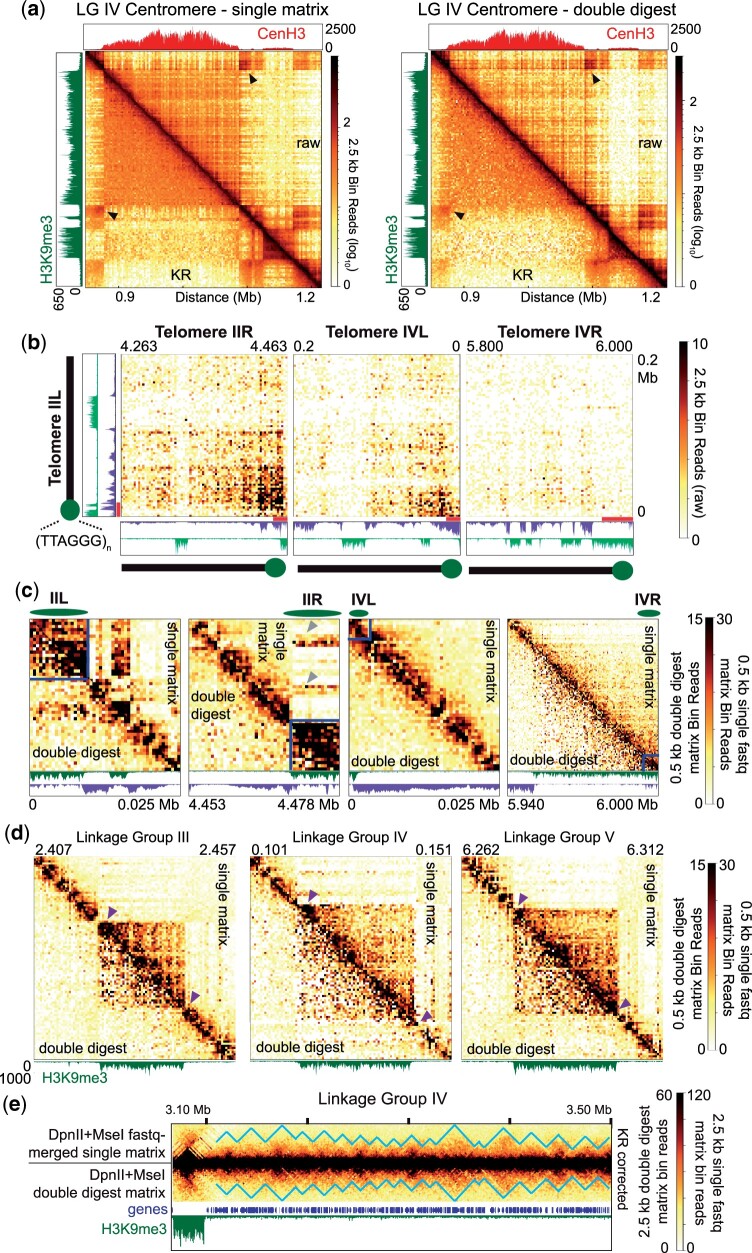
Characterization of individual chromatin region topology within the Neurospora genome. a) Heatmaps displaying the raw (above diagonal) and Knight-Ruiz (KR) corrected read count (below diagonal) of interactions at and surrounding the LG IV centromere at 2.5 kb bin resolution. The heatmap to the left uses the single in situ Hi-C matrix merged from *Mse*I and *Dpn*II fastq files (with 106M valid reads), while the right heatmap uses the DpnII and MseI double digest contact matrix. Arrowheads highlight pericentromeric interactions. CenH3 and H3K9me3 ChIP-seq enrichment tracks presented above and to the left of the image, respectively. b) Heatmaps displaying the raw read count of contacts between subtelomere IIL, and subtelomeres IIR, IVL, and IVR at 2.5 kb bin resolution in the DpnII and MseI double digest contact matrix. Subtelomere schematics (below and left) and lines indicate positions of apparent subtelomeres marked by H3K9me3; telomeres consist of ∼20 repeats of 5′-TTAGGG-3′, averaging ∼120 bp, at chromosome ends (Wu *et al.* 2009). H3K9me3 and H3K27me2/3 ChIP-seq enrichment tracks presented below and to the left at same *y*-axis scales as in Fig. 3a, c). KR-corrected heatmaps displaying the contacts extending 25 kb (or 60 kb for IVR) of 4 individual chromosome ends at 500 bp bin resolution using the single in situ Hi-C matrix merged from *Mse*I and *Dpn*II fastq files (giving 106M valid reads; above diagonal) or the *Dpn*II/*Mse*I double digest contact matrix (below diagonal). Lines and ovals (above) denote strong subtelomeric contacts as possible structures, which include the absolute telomeric sequences. H3K9me3 and H3K27me2/3 ChIP-seq enrichment tracks presented below at same *y*-axis scales as in Fig. 3a. Arrowheads show regions on heterochromatic LG IIR subtelomeric regions that interact with euchromatin in the single DpnII and MseI merged matrix that gives 106M valid reads. d). Knight-Ruiz (KR) corrected heatmaps displaying contacts of 3 interspersed heterochromatic regions at 500 bp bin resolution using the single in situ Hi-C matrix merged from *Mse*I and *Dpn*II fastq files (with 106M valid reads; above diagonal) or the *Dpn*II/*Mse*I double digest contact matrix (below diagonal). Position of each region indicated above, and H3K9me3 ChIP-seq enrichment track presented below. Arrowheads highlight dense globules either internal or immediately proximal to heterochromatic regions that may limit heterochromatin spread. e). KR-corrected in situ Hi-C heatmaps of contacts of a ∼500 kb euchromatic region of LG IV at 2.5 kb bin resolution using the single in situ Hi-C matrix merged from *Mse*I and *Dpn*II fastq files (above) or the *Dpn*II/*Mse*I double digest contact matrix (below). Triangles outline possible local euchromatin clusters observed in the KR-corrected matrix data. Gene and H3K9me3 ChIP-seq enrichment tracks shown below heatmap.

Using both the merged, single fastq and double digest datasets, we examined major intra- and interchromosomal contacts of silent regions, including centromeres, subtelomeres, and interspersed heterochromatic regions, to characterize their topology at a high resolution. Centromeric chromatin (“centrochromatin”), enriched with the centromere-specific histone variant CenH3 (Smith *et al.* 2011), strongly interacts, and is random and self-contained (Fig. 5a, Supplementary Fig. 25). All centrochromatin contacts present equally, especially in KR-corrected heatmaps, with a hierarchical compaction emanating from CenH3-containing nucleosomes. Most centromeric DNA is occluded from other DNA, apart from nearby euchromatic loops that contact presumably surface-exposed centromeric chromatin. Flanking pericentromeric regions, which each have robust interactions, strongly associate—implying a folded structure (Fig. 5a, black arrowheads, Supplementary Fig. 25). While centromeric nucleosomes containing CenH3 are still marked with H3K9me3, centromeres minimally associate with neighboring constitutive heterochromatic regions, and no interactions with H3K27me2/3-marked facultative heterochromatin occur. Apart from strong interchromosomal centromeric contacts (Figs. 2b and 3b, Supplementary Figs. 11, 17, 19, and 22), the H3K9me3/CenH3-marked centromeres are essentially segregated from other silent loci, supporting previous observations (Galazka *et al.* 2016; Klocko *et al.* 2016).

At chromosome termini, the subtelomeres—defined as ∼100–300 kb regions that extend beyond the repetitive telomeric DNA that are enriched with both H3K9me3 and H3K27me2/3 (Jamieson *et al.* 2013; Klocko *et al.* 2016)—strongly associate but segregate from centromeres (e.g. Figs. 2a, 3a, and 4a). The strongest contacts between the left and right subtelomeres of individual chromosomes, as well as interchromosomal subtelomere contacts, overlap H3K27me2/3 enriched regions, as evident in raw and KR-corrected contact heatmaps of telomere pairs (Fig. 5b, Supplementary Figs. 26–28); the strength of the individual binary pairings varies, implying an unknown organizational system [Fig. 5b, compare contacts of right (R) subtelomere of LG II and left (L) and right subtelomeres of LG IV with the left subtelomere of LG II; Supplementary Figs. S26-S28]. In contrast, individual subtelomeres have extensive contacts at, and extending from, the telomere across H3K9me3-enriched chromatin: several subtelomeres, including LG IIL, strongly contact neighboring constitutive heterochromatic regions (Fig. 5c, LG IIL). Other subtelomeres contact nearby euchromatin (e.g. LG IIR, gray arrowheads) or reside in larger heterochromatic domains (e.g. LG IVL/R; Fig. 5c).

Smaller, interspersed H3K9me3-rich constitutive heterochromatic regions have increased contacts that are occluded from facultative heterochromatin or euchromatin (Fig. 5d). The majority of these regions are flanked by extensive contacts across ∼two to five 500 bp bins at 1 or both ends of the H3K9me3 enriched region (Fig. 5d, purple arrowheads), suggesting the heterochromatic boundary associates with internal heterochromatin. A set of 100 randomly selected interspersed heterochromatic regions from all 7 chromosomes were inspected for increased heterochromatin/euchromatin boundary associations: 73 silent chromatin regions show increased interactions on both sides, and 94 silent regions have at least 1 side with increased contacts, possibly to prevent heterochromatin spread. All told, gene-poor, constitutive heterochromatic regions stochastically fold and strongly associate in the silent (B) compartment; inside this compartment, centromeres are segregated from other silent heterochromatic regions.

To examine euchromatin folding, we plotted nonlog transformed KR-corrected heatmaps in both our merged, single fastq and double digest in situ Hi-C datasets to highlight off-diagonal structures. We observe that euchromatin forms “globules” that are approximately 20–40 kb in size (Fig. 5e, left; blue line highlights globule boundaries), suggesting the chromatin fibers in active genic regions locally compact into small loops, highlighting a hierarchical organization to fold gene rich Neurospora DNA into the active “A” compartment in the center of the nucleus.

### Acetylated or methylated euchromatic genes associate with constitutive heterochromatin for gene regulation

**Fig. 6.**
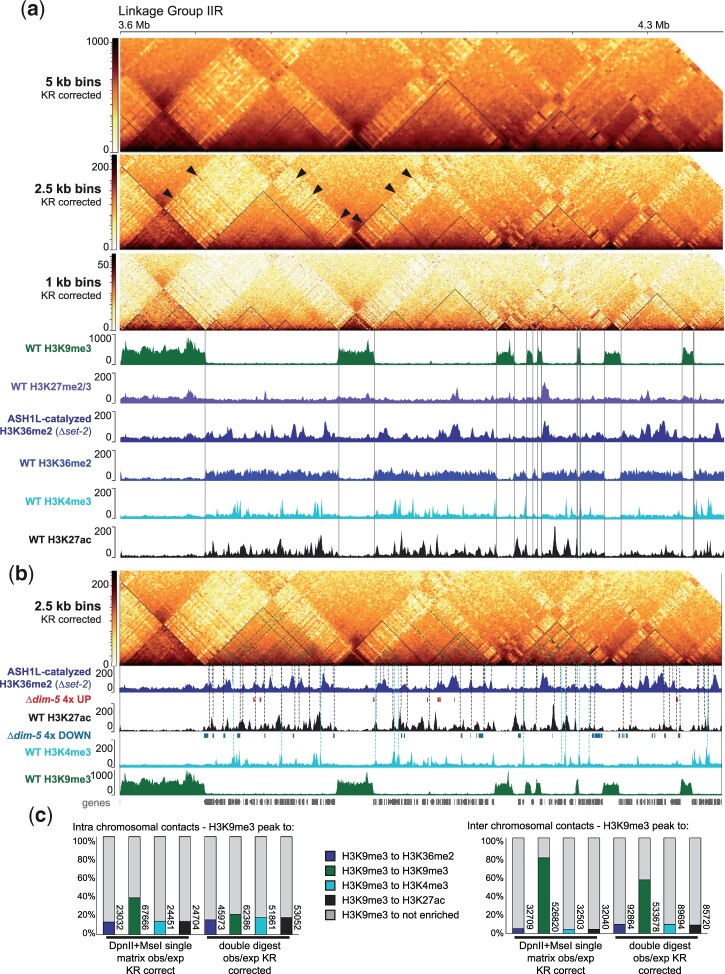
Long-range interactions between constitutive heterochromatin and individual euchromatic regions enriched with specific histone marks. a). KR-corrected Hi-C heatmaps of genomic interactions within the ∼700 kb region of the terminal right arm of LG II at progressively higher bin resolutions (5, 2.5, and 1 kb) aligned with the indicated ChIP-seq enrichment tracks and plotted on a log_10_ scale using the single in situ Hi-C matrix merged from *Mse*I and *Dpn*II fastq files (giving 106M valid reads). Triangle lines within the Hi-C heatmaps are the program-calculated Topologically Associated Domains (TADs), while the vertical lines originating from the 1 kb heatmap highlight the borders of the H3K9me3-marked constitutive heterochromatin domains. Arrowheads mark examples of euchromatic genes interacting with H3K9me3-marked heterochromatin. b). Identical 2.5 kb bin resolution KR-corrected Hi-C heatmap and 3 ChIP-seq enrichment tracks presented in A, and IGV images of bed files displaying genes that are 4-times increased or decreased in their expression in Δ*dim-5* strains. Dashed lines highlighting individual constitutive heterochromatin-euchromatic region interactions, colored as in panel (a): the 45° interactions originating from H3K9me3 regions are colored identically to the H3K9me3 ChIP-seq enrichment track, while the corresponding vertical lines ending in ChIP-seq enrichment tracks colored per tracks; multiple lines for each interaction between heterochromatin and euchromatin and different peaks of enrichment are shown. c). Graph of in situ Hi-C contact quantification across an observed vs expected 2.5 kb matrix (to normalize for genomic distance), scaled as the % of total contacts, with contacts originating at WT H3K9me3 enriched peaks and ending at one of the 4 possible enriched marks, colored as in A, or a region that is not enriched (see *Materials and Methods*). Only interchromosomal contacts log_2_ ≥ 3.5 and intrachromosomal contacts log_2_ ≥ 2.5 were counted. Numerical values of the interactions between WT H3K9me3 and either ASH1-catalyzed H3K26me2, WT H3K4m3, or WT H3K27ac are provided to the right of each bar; 664,816 total contacts originate in H3K9me3 in the single fastq matrix while 946,655 total contacts originate in H3K9me3 in the double digest. Bar graphs of the terminal enriched region are colored as the ChIP-seq tracks in (a).

To examine the topology of euchromatin for TAD-like structural elements, we used the hicPlotTads program to examine Neurospora euchromatin in our single fastq, merged in situ Hi-C dataset at increasingly higher resolutions. This program defined several TADs internal to euchromatic domains (Fig. 6a, thin line overlay), although visual inspection showed that the predicted TAD boundaries are most likely incorrect, as euchromatin outside of predicted TAD boundaries still strongly associates with TAD-internal euchromatin (Fig. 6a). As computational TAD prediction can vary (Zufferey *et al.* 2018), it is likely that these TAD boundaries were improperly called. We hesitate to argue that the Neurospora genome is organized by true TAD-like structures, considering the definition and establishment of TADs (see Discussion). However, this failed TAD prediction did have an overall positive outcome.

As we generated hicPlotTads output files, we displayed several published ChIP-seq tracks beneath high-resolution Hi-C contact heatmaps to glean information about the underlying euchromatin composition. We were struck by the presence of thin interactions between H3K9me3-marked constitutive heterochromatin and nearby euchromatin (Fig. 6a; black arrowheads). These euchromatic-heterochromatic interactions are observed at all resolutions examined but are best displayed at higher resolutions (≤2.5 kb bins), suggesting only small segments of euchromatic DNA associate with heterochromatin (Fig. 6a). Closer examination showed that interacting euchromatin is enriched for histone post-translational modifications, including the active marks H3K27ac and promoter-specific H3K4me3, as well as the repressive H3K36me2 catalyzed by ASH1 (Fig. 6b, dashed lines; here, Δ*set-2* H3K36me2 ChIP-seq elucidates the ASH1-specific H3K36me2, since two H3K36 methyltransferases are present in Neurospora, Bicocca *et al.* 2018). WT H3K27me2/3 minimally interacts with constitutive heterochromatin, and the density of WT H3K36me2 prevents correlational analysis (Fig. 6a). Quantification of the 2.5 kb bins in both the distance-normalized, single fastq and double digest datasets showed that ∼15% of all H3K9me3-enriched bins form intra- and interchromosomal contacts with euchromatin enriched for H3K27ac, H3K4me3, and ASH1-specific H3K36me2, although H3K9me3 to H3K9me3 contacts were the most prevalent (Fig. 6c).

To assess whether these topological contacts may influence transcription, we reanalyzed published poly-adenine RNA-sequencing datasets from WT and Δ*dim-5* strains (Klocko *et al.* 2016). Here, if euchromatic genes require contact with H3K9me3-marked silent chromatin for proper expression, loss of H3K9me3 would alter gene expression patterns. We observe multiple genes that form contacts with permanently silent chromatin and are enriched with histone post-translational modifications that have drastically altered (4-times) gene expression (Fig. 6b). Specifically, many genes enriched with H3K27ac or H3K4me3 that associate with silent chromatin are downregulated while several other heterochromatin-contacting genes with ASH1-catalyzed H3K36me2 are upregulated (Fig. 6b). We conclude that small euchromatic regions enriched for active or repressive histone modifications associate with constitutive heterochromatin for proper gene expression, possibly revealing a novel fungal mechanism for regulation of gene expression through chromatin topology.

## Discussion

**Fig. 7.**
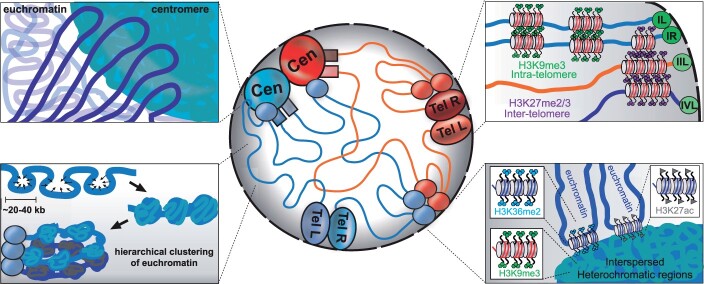
Model of genome topology of *N. crassa*. *Center*: general model of interactions of silent genome features within the nucleus of a wild type strain of *N. crassa*; adapted from Galazka *et al.* (2016). *Boxes*: novel genomic interactions identified in this work. Clockwise from the top left: euchromatic contacts on the bundled heterochromatic centromere; specific heterochromatic interactions of intra- and intertelomeric contacts; euchromatic genes enriched for acetylation or ASH1L-catalyzed H3K36me2 contact H3K9me3-marked constitutive heterochromatic regions for the regulation of gene expression; euchromatin is initially packaged into globules of ∼20–40 kb in size that are stacked into “Regional Globule Clusters”: euchromatic loops organized by association of heterochromatic regions.

Here, we characterize the genome topology of the filamentous fungus *N.* *crassa* at a high-resolution with 2 comprehensive Hi-C datasets: one that merges fastq files of Hi-C datasets independently generated by restriction enzymes monitoring euchromatin (*Dpn*II) and heterochromatin (*Mse*I), and a double digest dataset using both *Dpn*II and *Mse*I, each essentially generated with most current Hi-C protocol (e.g. Hi-C 3.0) (Lafontaine *et al.* 2021). These datasets show the Neurospora chromosome conformation in exquisite detail, unveiling principles of fungal genome topology (Fig. 7). Both datasets equally represent the conformation of euchromatin, suggesting gene contacts are accurate, which will support future gene regulation studies. However, heterochromatin-specific interactions appear more pronounced in the merged, single fastq Hi-C contact matrix—possibly, the *Dpn*II/*Mse*I double digest subtly over-represents rarer euchromatin-heterochromatin contacts and slightly depletes biologically relevant long-range contacts between heterochromatic regions; the latter is readily apparent in KR-corrected *Dpn*II or raw *Mse*I contact matrices. We slightly favor the merged, single fastq dataset, as it has more valid Hi-C interactions and therefore a greater depth of genomic contacts; we believe this dataset accurately displays genome topology within individual compartments (e.g., within heterochromatic or euchromatic regions), given the similarities of this dataset to the double digest contact matrix, as well as our computational experiment that shows excellent correlation between merged combinations of sampled *Dpn*II and *Mse*I replicates. However, our merged, single fastq dataset most likely underestimates contacts between euchromatic and heterochromatic compartments: upon KR correction, the underlying biases produced by the restriction enzyme used to generate that dataset are multiplicative (Yaffe and Tanay 2011; Imakaev *et al.* 2012), but when these datasets are combined (*e.g.*, fastq files are merged), the inherent biases of each dataset, within the merged dataset, become additive upon KR correction. This means cross-compartment contacts appear weaker in the single fastq, merged Hi-C matrices. While the overall fungal topology can be presented by merging fastq files of independently generated *Dpn*II and *Mse*I datasets, use of either *Dpn*II or *Mse*I independently in a single Hi-C experiment can monitor the contacts within a specific chromatin compartment in the Neurospora nucleus, an excellent advantage for the use of Neurospora in genome topology studies. Specifically, *Mse*I highlights contacts between heterochromatic regions >1 Mb apart while *Dpn*II captures more intra-compartmental euchromatic contacts, despite these restriction enzymes having identical digestion frequencies (e.g. *Dpn*II and *Mse*I both have recognition sites every 4^4^, or 256, bases). This is in contrast to higher organisms, such as humans, where completely different chromosome capture protocols, using different digestion enzymes (e.g. micrococcal nuclease), are needed to monitor short range (loops) or long range (compartments) structural features (Akgol Oksuz *et al.* 2021). Thus, our independent *Dpn*II and *Mse*I datasets are superb resources for future work to assess the contribution of individual genes for roles in forming the normal conformation of active or silent chromatin. In fact, our *Mse*I dataset allowed for the correction of the Neurospora reference genome, although numerous gaps are still present in our reported genome version (nc14), which would require long-read (e.g. PacBio) sequencing to fill. Considering that all our Hi-C libraries were generated with the latest in situ protocol to ligate contacting DNA in the nucleus (Rao *et al.* 2014; Tanizawa *et al.* 2017), we believe that our data are of high quality and effectively capture valid DNA contacts across the Neurospora genome. Previous work reports high-quality metazoan datasets show chromosomal territories with few interchromosomal contacts (Nagano *et al.* 2015). However, chromosome territories in fungi are controversial (Haber and Leung 1996; Lorenz *et al.* 2002; Liti *et al.* 2009; Zimmer and Fabre 2011), as fungal chromosomes minimally isolate, and substantial interchromosomal contacts are observed despite application of the in situ Hi-C protocol (Tanizawa *et al.* 2017). Indeed, a recent publication on the genome architecture across the Tree of Life highlights distinctions in topology between fungi and metazoans, with fungi exhibiting independent bundling of centromeres and telomeres (Hoencamp *et al.* 2021). Altogether, we suggest 1 measure of fungal Hi-C dataset quality is reduced mitochondria contacts, due to the isolation of nuclear and mitochondrial genomes in different organelles (Akgol Oksuz *et al.* 2021). By these standards, our presented fungal Hi-C data are of exceptional quality, given the few mitochondrial ligation products observed, and are consistent with the formation of weak territories with substantial interchromosomal, euchromatic contacts for the 7 Neurospora chromosomes. An exciting, unexplored possibility is that interchromosomal contacts regulate gene expression.

Our in situ Hi-C data allows us to make several general conclusions about fungal genome organization: heterochromatin is segregated from euchromatin, yet associations between heterochromatic regions mediate euchromatin looping; heterochromatic regions have stochastic internal contacts; heterochromatic histone marks correlate with the topology of some genomic features (*e.g.*, H3K9me3-marked silent regions are most often stochastically organized); and euchromatin enriched for certain histone marks associates with constitutive heterochromatin to impact gene expression. Our data are consistent with the compartmentalization of eukaryotic genomes into euchromatic (A) and heterochromatic (B) compartments (Lieberman-Aiden *et al.* 2009; Rao *et al.* 2014), which supports microscopic observations of the segregation of heterochromatin at the nuclear periphery while euchromatin has a more central localization in the nucleus (Franke 1974; Speicher and Carter 2005; Zuleger *et al.* 2011; Padeken and Heun 2014; Falk *et al.* 2019); this subnuclear architecture supports our hypothesis that KR-correction of *Mse*I-derived matrices excludes valid bundles of smaller, interspersed heterochromatic regions. Further, our in situ Hi-C data confirm microscopic observations in Neurospora showing the 7 centromeres, labeled with infrared fluorescent protein-tagged CenH3, cluster into a single focus localized to the nuclear membrane, yet these centromeres are segregated from telomeres, which present as 2–4 telomere clusters at the nuclear periphery when labeled with green fluorescent protein-tagged TRF1 (Galazka *et al.* 2016; Klocko *et al.* 2016). This Rabl-like chromosome organization observed in Neurospora is seen in other fungi, including *Epichloë festucae*, species of the *Verticillium* clade, *Agaricus bisporus* (Common mushroom), *Saccharomyces cerevisiae*, and *Schizosaccharomyces pombe* (Duan *et al.* 2010; Mizuguchi *et al.* 2014; Tanizawa *et al.* 2017; Winter *et al.* 2018; Seidl *et al.* 2020; Hoencamp *et al.* 2021), highlighting the importance of heterochromatic centromeres and telomeres for fungal genome topology. In contrast, the active, gene-rich euchromatic DNA in Neurospora is packaged into globules that are ∼20–40 kb in size—smaller than human globules (Rao *et al.* 2014, 2017) but consistent with globules in yeast (Mizuguchi *et al.* 2014). Cohesin forms these globules in other species, as enrichment of the cohesin subunit Rad-21 occurs at globule boundaries, and loss of Rad-21 depletes loops (Mizuguchi *et al.* 2014; Rao *et al.* 2017). Given the conservation of cohesin from fission yeast to humans, cohesin likely forms globules in Neurospora, and mutations in the Neurospora *rad-21* gene may elucidate local globule folding.

To structurally organize active DNA in the Neurospora genome, euchromatic globules are hierarchically packaged into layered “Regional Globular Clusters”; we suggest this term to distinguish the euchromatic structural elements in fungi from TADs in higher eukaryotes, and to clarify a previous report that Neurospora has TADs (Galazka *et al.* 2016). In a Regional Globule Cluster, euchromatin encompassing several hundred thousand basepairs of DNA and bordered by constitutive heterochromatin systematically loops upon the bundling of this flanking silent chromatin. Regional Globule Clusters are also observed in lower resolution Hi-C datasets (Galazka *et al.* 2016; Klocko *et al.* 2016). One might consider Regional Globular Clusters as primitive fungal TADs, but they certainly are not analogous to higher eukaryotic TADs. Considering the formal definition of Megabase-sized TADs (Dixon *et al.* 2012; Nora *et al.* 2012; Sexton *et al.* 2012; Szabo *et al.* 2020), true TADs have few long-range contacts beyond their boundaries. Our data shows that the smaller Regional Globular Clusters are layered and have extensive interlayer contacts, suggesting that higher eukaryotic proteins isolating external contacts from TADs, such as condensin II (Hoencamp *et al.* 2021), are not present in Neurospora.

Eukaryotic genomes are also organized by chromatin loops that average ∼200 kb in size (Rao *et al.* 2014). Higher eukaryotes encode the protein CTCF for loop formation (Yusufzai *et al.* 2004): each CTCF monomer binds a convergently oriented 17 basepair asymmetric DNA sequence, and CTCF dimerization forms the loop base (Rao *et al.* 2014); binding site choice for CTCF dimerization allows dynamic loop formation (Guo *et al.* 2015). Loops in humans are evident in Hi-C heatmaps as an enriched long-range contact at the intercept of 2 CTCF binding sites; enrichment is lost upon targeted CTCF degradation or binding sequence alteration (Rao *et al.* 2014; Guo *et al.* 2015; Nora *et al.* 2017), and CTCF is necessary to insulate promoters from aberrant enhancer/silencer contacts (Flavahan *et al.* 2016). The Neurospora genome does not contain the asymmetric 17 basepair CTCF binding sites, nor does it encode a CTCF homolog, which explains the lack of loop anchor enrichment foci here and in previous Neurospora Hi-C datasets (Galazka *et al.* 2016; Klocko *et al.* 2016) However, it is conceivable that constitutive heterochromatin takes the place of CTCF in fungi. In Neurospora, the heterochromatic regions interspersed throughout the genome would act as an “anchor” at the base of a euchromatic Regional Globular Cluster in a manner analogous to CTCF (Rao *et al.* 2014), although the dynamics of Regional Globular Clusters would be restricted to the subtle differences in the bundling between heterochromatic regions. Perhaps the seemingly unnecessary interspersed heterochromatic regions in the Neurospora genome are retained during evolution to structurally organize fungal chromosomes through formation of Regional Globular Clusters. One open question is if Regional Globular Clusters (or TADs) in any species have “epigenetic memory” for propagation across generations. In mammals, TADs are lost during mitosis and reform early in G1, implying TADs do not have any inherent epigenetic memory; it has been proposed that other factors, including histone post-translational modifications, reassemble TADs (Naumova *et al.* 2013). Perhaps in Neurospora, constitutive heterochromatic regions, which display epigenetic inheritance and spatially interact in the nucleus (Selker *et al.* 2003; Lewis *et al.* 2009; Galazka *et al.* 2016), are a simple mechanism to rapidly reform Regional Globular Clusters—and genome topology—during cell cycle progression. It is possible that liquid-phase separation of heterochromatin, as reported for human HP1α (Larson *et al.* 2017; Larson and Narlikar 2018) and proposed for genome compartmentalization (Falk *et al.* 2019) facilitates constitutive heterochromatin interactions. However, in Neurospora, loss of H3K9me3 (in a Δ*dim-5* strain lacking the single H3K9 methyltransferase) or HP1 minimally impacts heterochromatin contacts during fungal growth (Tamaru and Selker 2001; Tamaru *et al.* 2003; Lewis *et al.* 2009; Galazka *et al.* 2016), even though HP1 is important for genome topology in higher eukaryotes, as this protein is necessary to reorganize silent chromatin in the Drosophila genome during early embryogenesis (Zenk *et al.* 2021). Thus, in fungi, another inherent property of heterochromatin must drive liquid-phase separation to establish genome topology.

Liquid-phase separation would explain our high-resolution observations of internal heterochromatin organization, as the stochastic, pattern-less nature of Hi-C contacts internal to centromeres, telomeres, and interspersed heterochromatic regions is consistent with both random and variable internal packaging. At chromosome ends, heterochromatin facilitates intra- and interchromosomal subtelomeric contacts: individual subtelomeres are compacted at H3K9me3-marked constitutive heterochromatin, but H3K27me2/3-marked facultative heterochromatin facilitates contacts between subtelomeres. Some subtelomeres are contained within larger H3K9me3 domains, suggesting subtelomeres are uniquely recognized, perhaps by dual repressive histone marks. In Neurospora, loss of H3K27me2/3 (in a Δ*set-7* strain lacking the single H3K27 methyltransferase) compromises genome topology, as subtelomere clusters have reduced association with the nuclear membrane (Klocko *et al.* 2016), but H3K9me3 loss does not reduce subtelomeric interactions (Galazka *et al.* 2016). Thus, organization of chromosome ends must occur through distinct mechanisms mediated by both constitutive and facultative heterochromatin. One possibility is that constitutive heterochromatin condenses individual subtelomeres through liquid phase separation (Larson *et al.* 2017) while other proteins—such as EPR-1 or PAS (McNaught *et al.* 2020; Wiles *et al.* 2020) and/or Shelterin (Mizuguchi *et al.* 2017), which recognize/modulate facultative heterochromatin and bind telomere repeats, respectively—manage interchromosomal subtelomere contacts. Future work examining the role of these proteins/complexes in genome topology could clarify this hypothesis.

Small euchromatic segments enriched with active or repressive histone post-translational modifications associate with H3K9me3-marked heterochromatin in our comprehensive Hi-C datasets. Specifically, the small euchromatic regions that topologically associate with constitutive heterochromatin are enriched with activating marks such as acetylation of H3K27 and the trimethylation of H3K4 (Zhu *et al.* 2019), as well as a repressive mark, H3K36me2, catalyzed by ASH1 (Bicocca *et al.* 2018); these cross-compartment interactions may be more prevalent in the merged, single fastq dataset images due to the disparate frequencies of *Dpn*II and *Mse*I sites (in euchromatin and heterochromatin, respectively), focusing the interactions between genes and heterochromatic regions when dataset biases become additive upon fastq merging (above). While many of these contacts occur between heterochromatin and euchromatin separated by a few thousand basepairs, these intercompartment interactions may represent another layer of gene regulation: for activation, peaks of H3K27ac or H3K4me3 may indicate active genes or promoters require neighboring heterochromatin for maximal transcription, as has been observed for the Neurospora methionine synthase gene *met-8* (Yang *et al.* 2014). Conversely, silent genes may associate with constitutive heterochromatin at the nuclear periphery to prevent aberrant transcription initiation in the active nucleus center. Many of the genes marked by ASH1L-specific H3K36me2 are minimally transcribed and are also tri-methylated at H3K27 (Bicocca *et al.* 2018), suggesting Neurospora utilizes several mechanisms for repression. However, we observe minimal H3K27me2/3 enrichment across H3K36me2-marked genes that associate with silent chromatin—perhaps the association of ASH1-targeted genes with H3K9me-marked heterochromatin allows rapid transcriptional activation, analogous to poised developmental genes marked by H3K4me3 and H3K27me3 in higher eukaryotes (Voigt *et al.* 2013). Currently, the underlying mechanism for euchromatin looping to silent chromatin is unknown; an active recruitment mechanism by unknown protein(s) and a passive association mechanism, similar to liquid droplet formation (Larson and Narlikar 2018), are both feasible. We cannot rule out that these euchromatic regions are also marked with a low level of H3K9me3, which would be bound by HP1 to facilitate oligomerization with HP1-enriched constitutive heterochromatin, to colocalize these regions for regulation. Consistent with this hypothesis, the promoter of the *frq* gene encoding a master regulator of the Neurospora circadian rhythm has low levels of H3K9me3 that cycle over circadian time (Ruesch *et al.* 2014). Still, proper gene transcription requires uncompromised heterochromatin: RNA-sequencing of a Δ*dim-5* (aka: Δ*kmt1*) strain, which lacks tri-methylation of H3K9, has numerous previously unexplained gene expression changes, including aberrant transcriptional activation and repression relative to a WT strain (Klocko *et al.* 2016). We show several genes that contact constitutive heterochromatin in a WT strain have altered expression upon H3K9me3 loss: some acetylated genes become repressed while H3K36me2-marked genes are activated in a Δ*dim-5* (Δ*kmt1*) strain, although the correlation is inexact; of course, a specific combination of histone marks would be required for proper gene regulation in WT cells. It is unclear if altered gene expression occurs because topological changes no longer form, or if some component of H3K9me3-marked chromatin is necessary for gene regulation despite these long-range contacts still forming. High-resolution Hi-C of Δ*dim-5* (Δ*kmt1*) or Δ*hpo* strains may distinguish between these hypotheses. In retrospect, the re-localization of H3K27me2/3 that occurs in a Δ*dim-5* (Δ*kmt1*) strain (Basenko *et al.* 2015; Jamieson *et al.* 2016) presumably would not reestablish constitutive heterochromatin to restore proper gene expression, but our data partially explains the fact that few transcriptional changes are observed in a Δ*set-7* (Δ*kmt6*) strain lacking H3K27me2/3 (Klocko *et al.* 2016), as constitutive heterochromatin—and any long-range association between genes and silent chromatin—still forms.

In summary, our high-resolution Neurospora Hi-C datasets identify a novel mechanism involving genome topology for controlling gene expression; further experiments should clarify how euchromatin-heterochromatin interactions impact transcriptional regulation. We maintain that high-resolution Hi-C datasets are valuable tools for fungal researchers to elucidate the role of chromatin topology on genome function in fungal systems modeling human genome architecture or within fungal pathogens.

## Data availability

All WT *N. crassa* strains are available upon request. All in situ Hi-C high-throughput sequencing data of the WT Neurospora genome have been deposited to the National Center of Biotechnology Information (NCBI) Gene Expression Omnibus (GEO) public repository under the accession number GSE173593. Supplementary figures and tables are available at figshare: https://doi.org/10.25387/g3.17138534
